# Purinergic Signaling in Endometriosis-Associated Pain

**DOI:** 10.3390/ijms21228512

**Published:** 2020-11-12

**Authors:** Carla Trapero, Mireia Martín-Satué

**Affiliations:** 1 Departament de Patologia i Terapèutica Experimental, Facultat de Medicina i Ciències de la Salut, Campus Bellvitge, Universitat de Barcelona, 08907 Barcelona, Spain; ctrapero@ub.edu; 2 Institut d’Investigació Biomèdica de Bellvitge (IDIBELL), Oncobell Program, CIBERONC, 08908 Barcelona, Spain

**Keywords:** endometriosis, ATP, adenosine, P2Y, P2X, ectonucleotidases, pain, inflammation, endometrium, CD73, CD39

## Abstract

Endometriosis is an estrogen-dependent gynecological disease, with an associated chronic inflammatory component, characterized by the presence of endometrial tissue outside the uterine cavity. Its predominant symptom is pain, a condition notably altering the quality of life of women with the disease. This review is intended to exhaustively gather current knowledge on purinergic signaling in endometriosis-associated pain. Altered extracellular ATP hydrolysis, due to changes in ectonucleotidase activity, has been reported in endometriosis; the resulting accumulation of ATP in the endometriotic microenvironment points to sustained activation of nucleotide receptors (P2 receptors) capable of generating a persistent pain message. P2X3 receptor, expressed in sensory neurons, mediates nociceptive, neuropathic, and inflammatory pain, and is enrolled in endometriosis-related pain. Pharmacological inhibition of P2X3 receptor is under evaluation as a pain relief treatment for women with endometriosis. The role of other ATP receptors is also discussed here, e.g., P2X4 and P2X7 receptors, which are involved in inflammatory cell–nerve and microglia–nerve crosstalk, and therefore in inflammatory and neuropathic pain. Adenosine receptors (P1 receptors), by contrast, mainly play antinociceptive and anti-inflammatory roles. Purinome-targeted drugs, including nucleotide receptors and metabolizing enzymes, are potential non-hormonal therapeutic tools for the pharmacological management of endometriosis-related pain.

## 1. Introduction

Endometriosis is an estrogen-dependent gynecological disease characterized by the presence of endometrial tissue, glands, and stroma outside the uterine cavity, including the ovaries, pelvic peritoneum, and gastrointestinal tract, among other locations. In fact, depending on the location and characteristics, there are three different subtypes of endometriosis: peritoneal, ovarian, and deep infiltrating endometriosis [1]. It is estimated that this debilitating disease affects around 10% of women of reproductive age, but the true prevalence is difficult to quantify due to its unspecific symptoms and the lack of non-invasive diagnostic techniques that complicates diagnosis and can sometimes lead to misdiagnoses. Moreover, around 20–25% of women remain asymptomatic [2,3,4]. Even though pathogenesis of endometriosis is uncertain, the most widely accepted theory is the retrograde menstruation, described by Sampson, that includes three events that have to occur for endometriosis to develop: (i) menstrual blood flow backwards into the pelvic cavity (retrograde menstruation); (ii) the presence of available cells in menstrual debris; and (iii) the ectopic establishment and growth of endometrial cells [5]. More research on endometriosis is needed to fill the gaps in our understanding of its pathogenesis so as to help develop new diagnostic and therapeutic tools.

Associated with endometriosis is a chronic inflammatory component necessary for the establishment and progression of endometriotic lesions and which is related with its main symptoms and signs [6,7,8,9]. The main clinical features defining this disorder are pain and infertility, with the most common symptoms being cyclic pelvic pain, dysmenorrhea (painful menstrual periods), dyspareunia (painful sexual relations), dysuria (painful urination), and dyschezia (painful defecation) [10]. Different types of pain have been described in endometriosis: nociceptive, inflammatory, neuropathic, and a mixture of all of them. Interestingly, the predominant type of pain, its intensity, and its cyclicity vary between patients without a clear correlation between the scope of endometriotic lesions and pain experience [11]. This turns endometriosis-associated pain into a complex symptom that is difficult to manage.

Current treatments, both pharmacological and surgical, are addressed to providing symptom relief and are mainly focused on complex pain management, without effective results for all patients [11]. Several studies are attempting to overcome these obstacles by identifying molecular targets to develop new therapeutic approaches to improve the quality of life of affected women.

In recent decades, a large body of data has been published on the role of purinergic signaling in different inflammatory pathologies and its possible use as therapeutic target. The purinergic system is the extracellular signaling with biological effects mediated by nucleotides, such as adenosine triphosphate (ATP), and nucleosides, such as adenosine, involved in a wide range of physiological and pathological inflammatory conditions and in pain generation and transmission [12]. The purinergic signaling complex of a cell is sometimes referred to as the purinome [13]. In recent years, several studies have shown the involvement of purinome elements in endometriosis, but few studies have assessed their role as therapeutic targets for the endometriosis-derived chronic pain.

In this review, we aim to highlight the role of purinergic signaling in the pathogenesis and pathophysiology of endometriosis-associated pain. This information may be useful in presenting the molecular mechanisms underlying endometriosis-associated pain and toward the development of novel pharmacological approaches for endometriosis treatment.

## 2. Overview of Purinergic Signaling

Extracellular ATP and adenosine are the main purinergic mediators, with multiple roles in physiology and pathophysiology. The release of endogenous nucleotides and nucleosides into extracellular space by different cells in response to cell injury, necrosis, apoptosis, or various mechanical and chemical stimuli represents the beginning of the purinergic signaling cascade, which eventually induces an inflammatory response [12,14].

Under physiological conditions, extracellular ATP concentrations are low (submicromolar levels). However, with the release of endogenous ATP under situations such as inflammation, there is a marked increase of these levels [15]. 

**Figure 1:**
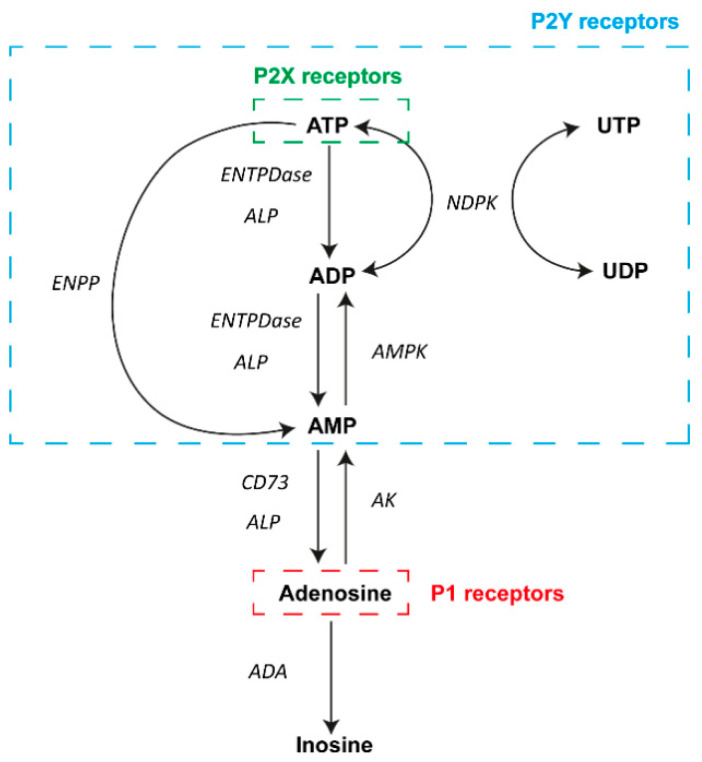
Diagram of the main elements of purinergic signaling. In the extracellular milieu, adenosine and nucleotides can activate P1 and P2 receptors on the surface of a wide variety of cell types. A number of different nucleotides can activate P2Y receptors (blue box), and ATP can also activate P2X receptors (green box). In contrast, adenosine actions involve the activation of P1 receptors (red box). Different enzymes are involved in the metabolism of adenosine and ATP in the process of achieving transient signaling. Abbreviations: adenosine triphosphate, ATP; adenosine diphosphate, ADP; adenosine monophosphate, AMP; uridine triphosphate, UTP; uridine diphosphate, UDP; ectonucleoside triphosphate diphosphohydrolase family, ENTPDase; alkaline phosphatase family, ALP; ectonucleotide pyrophosphatase/phosphodiesterase family, ENPP; ecto-5′-nucleotidase, CD73; nucleoside diphosphate kinase, NDPK; AMP-activated protein kinase, AMPK; adenosine kinase, AK; adenosine deaminase, ADA.

To avoid sustained ATP signaling and the adverse effects of increased extracellular ATP levels, ectonucleotidases act extracellularly by degrading ATP into adenosine. Ectonucleotidases are specialized nucleotide-hydrolyzing enzymes, broadly expressed at the cell surface of many tissues, which, acting alone or sequentially, control nucleotide and nucleosides levels in the extracellular milieu (Figure 1). Four families of ectonucleotidase have been described: (i) the ectonucleoside triphosphate diphosphohydrolase (ENTPDase) family (also known as CD39 family), with NTPDase1 (CD39), -2, -3, and -8 as plasma membrane-bound members, which hydrolyze extracellular ATP to adenosine diphosphate (ADP), and ADP to adenosine monophosphate (AMP); (ii) the ectonucleotide pyrophosphatase/phosphodiesterase (ENPP) family, which converts ATP to AMP and inorganic pyrophosphate (PPi); (iii) the 5′-nucleotidase family, with only one membrane-bound member, the ecto-5′-nucleotidase, also known as CD73, which hydrolyzes AMP to adenosine; and (iv) the alkaline phosphatase (ALP) family, able to hydrolyze adenine nucleotides and pyrophosphate, releasing inorganic phosphate (Pi) [12,16].

Adenosine also has important biological functions. In general, adenosine has been linked mainly to an anti-inflammatory effect. There are two enzymes responsible for adenosine metabolism: adenosine deaminase (ADA) and adenosine kinase (AK). ADA is a cytoplasmic enzyme but also an ectoenzyme that regulates intra- and extracellular adenosine levels, catalyzing the adenosine deamination yielding inosine [12]. As ectoenzyme, ADA is expressed as a soluble form or as membrane-associated enzyme-forming complexes with CD26/dipeptidyl peptidase IV in humans. AK is a cytosolic enzyme that catalyzes the phosphorylation of intracellular adenosine to AMP [12].

Once in the extracellular microenvironment, adenosine and nucleotides can activate two different purinergic receptor families, P1 and P2, respectively, which are widely and differentially expressed in the surface of most cells. Adenosine binds to P1 receptors, which are G protein-coupled receptors classified into four subtypes: A_1_, A_2A_, A_2B_, and A_3_ [17]. These receptors mainly act via adenylate cyclase (AC) activity, modulating cyclic AMP (cAMP) production. The stimulation of the A_2A_ and A_2B_ receptors induces activation of AC, increasing second-messenger cAMP levels. In contrast, the activation of the A_1_ and A_3_ receptors inhibits AC, causing a decrease in cAMP production [17]. Furthermore, A_3_ and A_2B_ receptors lead to the activation of phospholipase C (PLC) and an increase in intracellular calcium levels [18,19]. Moreover, A_1_ receptor is involved in the opening of K^+^ channels [18,19]. Cation mobilization triggered by adenosine receptors leads to transmission and modulation of pain. In addition, adenosine receptors stimulate mitogen-activated protein kinases (MAPK), regulating growth and proliferation, apoptosis, necrosis, and inflammation, essential for the development of endometriosis [20].

In addition, P2 receptors are nucleotide-selective and include P2X and P2Y receptor subtypes. The ionotropic P2X receptors, comprising seven subtypes (P2X1–P2X7), are ligand-gate ion channels strictly activated by extracellular ATP to mediate K^+^ efflux, and Na^+^ and Ca^2+^ influx, which mainly mediate short-term (acute) purinergic signaling, and which play an important role as mediators of fast excitatory neurotransmission in the central and peripheral nervous system [17,21]. The metabotropic P2Y receptors are G protein-coupled receptors that activate PLC-β (P2Y_1_, P2Y_2_, P2Y_4_, P2Y_6_, and P2Y_11_) or inhibit AC (P2Y_11–14_). The P2Y receptors have distinctive nucleotide preferences and, based on this, they can be divided in three groups: (i) adenine nucleotide-preferring receptors, mainly responding to ATP (P2Y_1_, P2Y_2_, P2Y_4_, and P2Y_11_) and ADP (P2Y_1_, P2Y_12_, P2Y_13_); (ii) uracil nucleotide-preferring receptors, activated by UTP (P2Y_2_, P2Y_4_, and P2Y_6_) and/or UDP (P2Y_6_ and P2Y_14_); and (iii) the P2Y_14_ nucleotide sugar-preferring receptor, responding to UDP sugars, such as UDP-glucose and UDP-galactose [17,22,23]. P1 and P2Y G protein-coupled receptors are predominantly involved in long-term (trophic) purinergic signaling, such as that found in remodeling, repair, and regeneration events in response to injury. P1 and P2Y receptors are involved in the regulation of proliferation, differentiation, motility, migration, and cell death [21], which are essential processes for the establishment of endometriotic lesions and the progression of endometriosis.

Correct expression and function of the purinome are essential to maintaining tissue homeostasis. Changes in ATP/adenosine balance and in the purinergic receptor activation state can alter the behavior of a wide range of cell types. These changes can trigger a pathological state or promote the progression of a disease, as may occur in endometriosis.

## 3. Purinergic Signaling in Eutopic and Ectopic Endometrial Tissue

Extracellular purines and pyrimidines play multiples roles in fertilization and embryo development [24,25]. This is made possible by different elements of the purinome, such as purinergic receptors and ectonucleotidases, present in both male and female reproductive organs [26,27,28,29,30,31,32,33]. 

Endometrium, the innermost layer of the uterus, contains the surface epithelium, the glandular epithelium, and a vascularized stroma. It is a dynamic tissue undergoing repetitive cycles of regeneration and degeneration in which a certain degree of inflammation is physiological. Purinergic signaling plays a role in both inflammation and reproduction and contributes to correct endometrial function. Therefore, it is not surprising that any change in purinergic signaling can alter uterine function and fertility in women, as happens in endometriosis. In addition, purinergic signaling is involved in the control of a number of cell events, such as cell proliferation, migration, and survival, but it is also seen in phenomena such as angiogenesis and fibrosis (reviewed in [34,35,36]), thus becoming a candidate pathway for playing a causative role in the pathogenesis and development of this disease.

Therefore, the characterization of the purinome elements of eutopic endometrium and endometriotic lesions is essential in order not only to elucidate the role of purinergic signaling in the pathogenesis, but also to identify the main symptoms and signs of endometriosis and new therapeutic targets. Moreover, knowledge of the microenvironment of ectopic lesions can improve understanding of the generation and neurotransmission of pain signals in endometriosis. We present below current knowledge on the expression of ectonucleotidases, the main elements of the purinome regulating the levels of nucleotides and nucleosides in eutopic and ectopic microenvironments.

### Ectonucleotidases in the Eutopic and Ectopic Endometria of Women with Endometriosis

Ectonucleotidases are hormone-sensitive enzymes that vary their expression in the endometrium throughout the menstrual cycle [26]. NTPDase2, NTPDase3, NPP1, NPP3, ALP, CD26, and CD73 are expressed by endometrial epithelial cells, while NTPDase1, NTPDase2, and CD73 have been detected in endometrial stromal cells [26]. Although the ectonucleotidases studied to date are mainly present in the endometrial epithelium, most of the changes detected in endometriosis occur in the stroma [37].

Changes in eutopic endometrium of women with endometriosis in comparison with the endometrium of women without the pathology have been detected [26,37]. Moreover, differences between the different types of endometriotic lesions have been described [37]. One notable change in endometriosis is the expression of NPP3. Several studies of NPP3 expression in endometrial tissue have localized the protein in epithelial cells, with changes along the cycle [26,38,39]. In endometriosis, however, NPP3 is found to be expressed by the stroma, as well as the epithelium, in both eutopic and ectopic endometrial tissues [37]. This de novo expression of NPP3 points to its use as a putative histopathological marker of the disease. It has to be noted that although there is no protein expression of NPP3 in the endometrial stroma of women without endometrial pathology, Boggavarapu et al. detected more than double the levels of NPP3 mRNA in the stroma compared to glandular compartment [40]. All of this points to post-translational regulation of protein levels that needs to be further studied. This increased expression of NPP3 in endometrial tissue coincides with the increased ectonucleotidase activity detected in the fluid content of ovarian endometriomas [41]. These results suggest an increase in ATP metabolism, with a concomitant increase in extracellular adenosine levels, as observed in some cancers, in which high levels of adenosine in the tumor microenvironment induce suppression of the local immune response [42,43]. Conversely, the detection of one hundred times higher levels of ADA in the contents of endometriomas refutes the idea of adenosine accumulation [44]. Moreover, the great decrease in, or even the total loss, of the CD39–CD73 axis in endometrial tissue in endometriosis further suggests that extracellular adenosine synthesis is rather limited. [37]. In fact, these data suggest a relation between extracellular ATP accumulation and the severity and progression of endometriosis, since the loss of CD39–CD73 is related to deep infiltrating endometriosis, the most severe form of the illness with high recurrence rates and a high level of associated pain [45,46]. 

The consequences of the increase in extracellular ATP levels and the subsequent activation of purinergic signaling through P2 receptors in endometriosis-associated pain are discussed throughout this review.

## 4. Involvement of Purinergic Signaling in Endometriosis-Associated Pain

### 4.1. Endometriosis-Associated Pain

Pain is recognized as the most common symptom and the primary reason for medical assistance in women with endometriosis. In fact, up to 80% of patients present chronic pain, the most common forms being dysmenorrhea, non-cyclical pelvic pain (chronic pelvic pain), dyspareunia, dysuria, and dyschezia [2]. Endometriosis-associated pain has, in turn, negative effects on women’s mental health, including anxiety and depression, thereby altering their quality of life and that of their loved ones [47,48]. For this reason, there is an urgent need to define the molecular mechanisms underlying endometriosis-associated pain to uncover therapeutic targets to minimize the suffering and raise the quality of life of affected women.

Endometriosis-associated pain is complex, and the underlying mechanisms seem to be related to the activation of the peripheral nervous system, involved in nociceptive (a response to a noxious stimulus), inflammatory (due to tissue damage and inflammatory response), and neuropathic (due to a lesion in somatosensory nervous system) pain, and central nervous systems, related with sensitization and hyperalgesia processes [49,50]. The stage of endometriosis, as classified by the American Society of Reproductive Medicine (ASRM), poorly correlates with the degree of pain or symptoms severity, thus hampering clinical management [11].

In recent years, many articles concerning endometriosis-associated pain and treatments for pain-relief have been published (reviewed in [10,11,49,51,52,53,54,55,56,57,58,59,60,61,62,63]); independently, the purinergic mechanisms involved in pain (reviewed in [18,64,65,66,67,68,69,70]) are also being studied. However, purinergic signaling in relation to endometriosis-associated pain has yet to be fully explored. In the following section, we look at the involvement of the purinergic signaling in pain, especially in peripheral, but also in central processes, in the context of endometriosis.

#### 4.1.1. The Pain Pathway

The neural process of pain starts with a peripheral noxious stimulus detected by the nociceptors on small diameter sensory afferent nerves (fibers Aδ and C) and its transduction into an electric signal. These neurons, which innervate viscera, have the cell bodies in the dorsal root ganglia (DRG) and reach the lamina I-II of the dorsal spinal cord. This information is transmitted along the spinal cord to the brain, where the unpleasant experience called pain is generated. Although multiple painful conditions have their origin in the sensitization and excitation of neurons, immune and glial cells also play key roles in the generation and maintenance of pain signaling [71,72]. Indeed, the pain perceived can be altered, amplified, or reduced by many molecules, including ATP, released from these non-neuronal cells through different mechanisms required for the transition from acute to chronic pain [72,73]. ATP is a peripheral mediator of pain involved in the initiation of this pain perception.

There is evidence that endometriotic lesions are innervated. This innervation is mainly sympathetic, and sensory nerve fibers play a pivotal role in endometriosis-associated pain [74,75,76,77,78]. The inflammation, concomitant to the initial establishment of an endometriotic focus outside the uterus, activates sensory afferent neurons innervating adjacent visceral structures, transmitting the noxious stimulus to the spinal dorsal horn and causing pain. Moreover, local inflammatory cells release neurotrophic factors encouraging the new innervation and cytokines that lead to the implantation of endometrial ectopic cells. Neuronal and non-neuronal cells of endometriotic lesions can release ATP that in turn regulates the action of these cells. For example, the release of ATP and neurotransmitters by afferent neurons also activates spinal glial cells, contributing to central sensitization and overstated pain. 

Endometriotic foci present cyclic proliferative and destructive phases similar to the endometrium. During the breakdown of a part of a lesion, high levels of ATP are released in the lesion microenvironment, acting as an acute danger signal on sensory nerve endings of the lesion. Moreover, this internal bleeding in the ectopic locations often leads to local inflammatory reactions that promote the inflammatory state and the release of molecules involved in the pain signaling pathway. In addition, ATP has the potential to modify the pain signaling by activation of pre- and post-synaptic P2 receptors. The two classes of P2 receptors are involved in pain: P2X receptors in short-term neurotransmission responses and P2Y receptors in the slow and continuous pain signaling.

The high levels of extracellular ATP in the ectopic milieu, together with the loss of the CD39–CD73 axis, turns purinergic signaling into a precious source of possible therapeutic targets for endometriosis-associated pain treatment. The role of ATP and adenosine in the pain signaling pathway in the context of endometriosis is reviewed in detail below.

#### 4.1.2. P2X Receptors in Primary Sensory Neuron: The Outset of Nociception

*P2X3 and P2X2/3 receptors*. P2X3 and P2X2/3 receptors are expressed in terminals of nociceptive fibers and in the sensory neurons of the central nervous system. Homomeric P2X3 receptors mediate transient nociceptive responses through rapidly desensitizing current, and heteromeric P2X2/3 receptors mediate sustained nociceptive responses through a slowly desensitizing current [79]. They mediate neuropathic pain, including inflammatory pain, in acute and chronic processes, and are involved in hyperalgesia and allodynia [80]. In fact, sensory neurons which express transient receptor potential vanilloid-1 (TRPV1) channels and/or P2X3 receptors are essential for the initiation and transduction of nociception and the signaling of pain. Both TRPV1 and P2X3 induce Ca^2+^ influx which activates a cascade of changes, including the phosphorylation of ion channels with the consequent increase in the excitability of sensory neurons. 

Estrogens upregulate the expression of both the cation channel TRPV1 and P2X3 receptors of nerve fiber terminals in endometriosis [81]. The implication of TRPV1 in endometriosis-associated pain is clear, but the precise role of P2X3 is still under study, although there is increasing evidence of its central role in the onset of pain sensation in endometriosis. Ding et al. detected the expression of P2X3 in endometriotic epithelial and stromal cells but also in the sensory nerve fibers within endometriotic lesions [82]. Moreover, they positively correlated the levels of P2X3 receptor in endometriotic lesions with the severity of pain [82].

The activation of P2X3 receptor in nerve fibers leads to the release of endogenous ATP via pannexin-1 hemichannels, triggering the activation of P2 receptors of sensory nerve fibers [83]. In addition, this ATP efflux can be enhanced by nerve growth factors [83]. Persistent extracellular ATP with the concomitant P2X3 and P2X2/3 receptor activation has been linked to the induction and the early maintenance phases of allodynia, a clinical manifestation of chronic pain present in endometriosis [84,85].

In rats, the induction of endometriosis produces thermal and mechanical hyperalgesia. Moreover, endometriosis causes the elevation of endogenous ATP content and P2X3 receptor expression in endometriotic and DRG tissues, which correlate with the severity of hyperalgesia in these animals [86]. In fact, the implication of ATP and P2X3 in endometriosis-associated pain was also confirmed, since administration of A-317491, a selective P2X3 receptor antagonist, attenuated endometriosis-associated pain in rats [87]. 

Study of the upregulation of P2X3 receptor in endometriosis-related pain showed ATP and ADP, but not UTP, as the effector molecules of this process [86]. In accordance with P2 receptor affinities, P2Y_1_, P2Y_12_, and/or P2Y_13_ receptors could be responsible for this upregulation. Ding et al. determined that P2X3 upregulation by ADP is mediated by the activation of the transcription factors ATF3 and AP-1 [86]. Moreover, based on the literature, they discussed the possible role of P2Y_1_ as the promoter of this pathway, but functional studies are needed to confirm this point [86]. 

Hence, extracellular ATP seems to have a close relationship with initiation, amplification, and maintenance of endometriosis-related pain [87]. In a persistent damaging inflammatory microenvironment, such as the one generally found in the peritoneum of women with endometriosis, high levels of inflammatory mediators are detected. Endometrial cells and immune cells in the ectopic lesions secrete different inflammatory mediators such as interleukin (IL)-1β, IL-6, and tumor necrosis factor alpha (TNF-α) [71,88], as well as growth factors such as vascular endothelial growth factor (VEGF), neurotropin nerve growth factor (NGF), and brain-derived neurotrophic factor (BDNF), often promoted by the presence of high estrogen levels [71,89,90,91]. These factors not only promote the inflammatory state and the aberrant neuroangiogenic milieu, but are also involved in processes that reduce the threshold of ion channels of sensory fibers, increasing their membrane excitability and the expression of receptors involved in nociception, thus favoring the activation of pain signaling pathway. These molecules promote the release of endogenous ATP during inflammation with the subsequent activation of P2X3 and P2X2/3 receptors, allowing Ca^2+^ influx and depolarization in nearby nociceptive fibers of the endometriotic foci and leading to the sensitization of sensory neurons, which send the pain message to the central nervous system. Therefore, ATP triggers the purinergic signaling cascade in nervous cells involved in pain symptoms. However, P2X3 activation by ATP as a potential action generator is only the beginning of ATP participation in the pain signaling pathway, as shown below.

The activation of the receptors tyrosine kinase A (TrkA), p75, and VEGFR2 in primary sensory neurons mediated by NGF and VEGF, secreted by endometriotic and immune cells, triggers the upregulation of P2X3, causing repeated neuronal sensitization by increased P2X3 receptor signaling and provoking a persistent sensation of pain [92,93]. Moreover, NGF plays a role in the production of the neuropeptide substance P (SP) and calcitonin gene-related peptide (CGRP), which sensitize the sensory nerve fibers, stimulate the immune system, cause the degranulation of mast cells, and promote fibrosis of the lesion through activation of their receptors neurokinin-1 receptor (NK1R) and calcitonin gene-related peptide receptor (CGRPR), respectively [94,95]. Moreover, the high levels of estrogens detected in the microenvironment of endometriotic lesions trigger the degranulation and secretion of NGF by mast cells [96]. Mast cell granules contain inflammatory mediators and neuro-sensitizing molecules including IL-1β, IL-6, TNF-α, and histamine, which, once released into the endometriotic milieu, encourage nerve sensitization and the inflammatory state [97].

#### 4.1.3. P2Y Receptors in Primary Sensory Neuron: The Modulation of Nociception

Primary sensory neurons also express P2Y receptors and their role is mainly pain modulation. P2Y receptors potentiate pain induced by chemical or physical stimuli via capsaicin-sensitive TRPV1 channels and facilitate the P2X receptor-mediated currents [98,99]. 

*P2Y_1_ and P2Y_2_ receptors.* Activation of P2Y_1_ and P2Y_2_ receptors is involved in the activation of TRPV1 channels of nociceptors. Although in humans it is not clear, in rats, upregulation of TRPV1 channel expression by P2Y_1_ receptors is mediated via p38/MAPK [100,101]. Therefore, it is probably the case that ATP and ADP are involved in short- and long-term effects in nociceptors. On the one hand, they can play a role in the modulation of Ca^2+^ influx that potentiates the sensitization of sensory neurons, while, on the other hand, they may be involved in the long-term nociceptor changes that produce hyperalgesia through the upregulation of TRPV1 channels. Moreover, as stated above, P2Y_1_ may also play a role in the upregulation of P2X3 receptor ion channel in endometriosis-associated pain [86]. In inflammation, P2Y_2_ receptor upregulation occurs in sensory neurons of inflamed tissue [98,102]. ATP (and UTP) stimulus on P2Y_2_ receptors activates TRPV1 channels [103]. This points to the contribution of ATP to chronic inflammatory pain, and therefore, endometriosis. 

#### 4.1.4. P2X4 and P2X7 in Macrophage–Nerve Interaction: The Base of Inflammatory Pain

**Figure 2:**
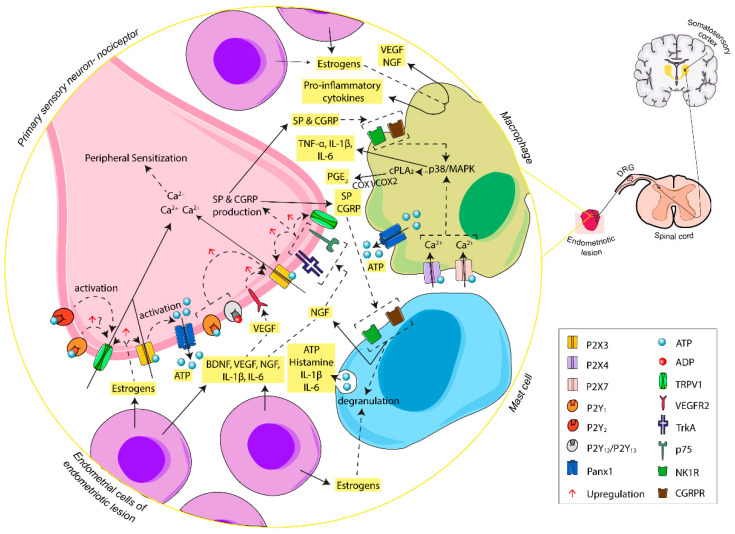
Schematic summary of the involvement of ATP, through activation of P2X and P2Y receptors, in the initiation of endometriosis-associated pain. In the endometriotic lesion, ATP, released by different cell sources, carries out Ca^2+^ influx via P2X3 receptor activation at the endings of primary sensory neurons, triggering a cascade of changes that increase the excitability of afferent sensory neurons. The activation of P2Y receptors potentiates the action of P2X3 receptor and TRPV1, triggering induction of nociception and the maintenance of overstated pain. Moreover, ectopic endometrial cells and inflammatory cells of the lesion release inflammatory mediators that boost nerve sensitization and promote the inflammatory state typical of women with endometriosis. Abbreviations: adenosine triphosphate, ATP; adenosine diphosphate, ADP; pannexin-1, Panx1; brain-derived neurotrophic factor, BDNF; neurotropin nerve growth factor, NGF; tyrosine kinase A receptor, TrkA; p75 neurotrophin receptor, p75; vascular endothelial growth factor, VEGF; vascular endothelial growth factor receptor 2, VEGFR2; interleukin-1 beta, IL-1β; interleukin-6, IL-6; tumor necrosis factor alpha, TNF-α; substance P, SP; neurokinin-1 receptor, NK1R; calcitonin gene-related peptide, CGRP; calcitonin gene-related peptide receptor, CGRPR; transient receptor potential vanilloid-1 channel, TRPV1; p38 mitogen-activated protein kinases, p38/MAPK; cytosolic phospholipase A2, cPLA_2_; prostaglandin E2, PGE2; cyclooxygenase-1 and -2, COX-1/COX-2; dorsal root ganglia, DRG.

As a consequence of the inflammatory process, large numbers of macrophages, mastocytes, and neutrophils are recruited in the endometriotic focus and macrophages infiltrate DRG [71,96,104]. Macrophages are among the most numerous immune cells in endometriotic lesions. They produce pro-inflammatory cytokines such as IL-1β, TNF-α, and IL-6, which intervene in the pain phenomena [105] and have a role in endometriosis-associated pain (reviewed in [71]). The activation of P2 receptors expressed by immune cells, such as P2X7 and P2X4 receptors in macrophages [106], allows the activation of the immune system via ATP and leads to the production of cytokines, thus maintaining the persistent inflammatory state. Moreover, the activation of macrophage P2X4 receptors is involved in the release of COX-dependent release of prostaglandin E2 (PGE2), mediated by cytosolic phospholipase A2 (cPLA2) [107]. PGE2 is involved in the sensitization of primary sensory neurons [107]. The interaction among endometrial cells, inflammatory cells, and peripheral sensory neurons at the ectopic foci, and the ATP-mediated molecular pathways, are represented in Figure 2.

#### 4.1.5. P2 Receptors in Activated Microglia: The Modulation of Pain Transmission

Endometriosis-associated pain is not only inflammatory but also neuropathic [82,108]. Nerve damage and persistent stimulation of peripheral fibers can lead to the secretion of inflammatory neurotransmitters and neuromodulators from nerve fibers, including ATP, which acts on glial cells. In the meantime, glial cells react, becoming the main source of neuroactive substances, including pro-inflammatory cytokines, trophic factors, and neurotransmitters (such as ATP), which regulate neuronal excitability and are fundamental to the transition from acute to chronic pain.

**Figure 3:**
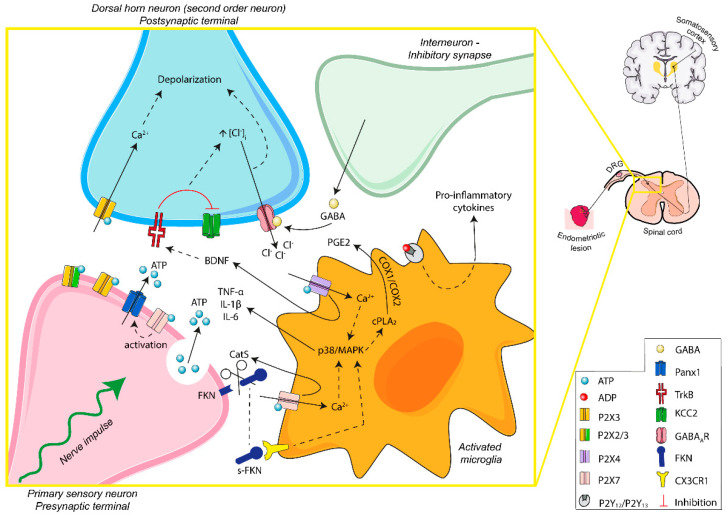
Schematic overview of ATP signaling in microglia–nerve interaction in endometriosis-associated pain. Persistent stimulation of peripheral fibers leads to the release of ATP into the synaptic space. The activation of P2X3 receptors of postsynaptic terminals induces excitability of dorsal horn neurons. Moreover, P2X4 and P2X7 receptors of activated microglia are involved in the secretion of neuroactive substances, contributing to the microglia–nerve interaction needed for central sensitization and modulation of the sensation of pain. ATP-mediated signaling is fundamental in the transition from acute to chronic pain, including endometriosis-related neuropathic and inflammatory pain. Abbreviations: adenosine triphosphate, ATP; adenosine diphosphate, ADP; pannexin-1, Panx1; brain-derived neurotrophic factor, BDNF; interleukin-1 beta, IL-1β; interleukin-6, IL-6; tumor necrosis factor alpha, TNF-α; gamma-aminobutyric acid, GABA; gamma-aminobutyric acid A receptor, GABA_A_R; p38 mitogen-activated protein kinases, p38/MAPK; cytosolic phospholipase A2, cPLA_2_; prostaglandin E2, PGE2; cyclooxygenase-1 and -2, COX-1/COX-2; fractalkine, FKN; soluble-fractalkine, s-FKN; cathepsin S, CatS; CX3C chemokine receptor 1, CX3CR1; tyrosine kinase B receptor, TrkB; potassium-chloride cotransporter 2, KCC2; dorsal root ganglia, DRG.

P2 receptors are present in the surface of activated spinal microglia and are involved in the recruitment and activation of microglia and the interaction between neurons and microglia (summarized in Figure 3). All of these play important roles in neuropathic pain, mainly in allodynia and hyperalgesia, and in neuroinflammation. 

Apparently, the activation of microglial P2 receptors, particularly P2X4 and P2X7, promotes neuronal excitability. Therefore, blocking microglia–neuron signaling must be considered as a possible therapeutic strategy for treating endometriosis-associated pain.

*P2X4 receptor*. The role of the P2X4 receptor in activated microglia in endometriosis-associated pain has not yet been studied, but P2X4 receptor involvement in neuropathic pain is clear. Following peripheral nerve injury, P24 receptor is expressed by the microglia of the dorsal horn [109]. The activation of P2X4 receptor causes Ca^2+^ flux and p38/MAPK activation, which promotes the synthesis and release of BDNF, a key molecule for maintaining pain hypersensitivity. ATP-mediated BDNF release from activated microglia, via its receptor TrkB, mediates the downregulation of the K^+^/Cl^−^ cotransporter KCC2 in dorsal horn neurons. KCC2 maintains the anion gradient necessary for the inhibitory actions of gamma-aminobutyric acid (GABA) through gamma-aminobutyric acid A receptor (GABA_A_R). KCC2 downregulation increases intracellular chloride levels, allowing the accumulation of anions in dorsal horn neurons. Meanwhile, GABA is released from inhibitory interneurons. GABA activates the GABA_A_R on dorsal horn neurons with the subsequent chloride outflow, causing the depolarization of the second order neurons. Hence, BDNF alters the chloride gradient of dorsal horn neurons, triggering a decrease in the inhibitory control of the GABAergic interneurons. Consequently, there is an increase in dorsal horn neuron excitability that enables low threshold information to gain access to nociceptive circuits. This evokes pain transmission and neuropathic pain [109,110,111]. In addition, P2X4 receptor, through p38/MAPK activation, leads to the synthesis and release of pro-inflammatory cytokines and increases the expression of COX, enhancing PGE2 levels, involved in pain-related inflammatory responses and dorsal horn neuronal excitability [112,113]. It is noteworthy, however, that P2X4 is not the only one. The activation of P2X7 (by ATP) and P2Y_12_ and P2Y_13_ receptors (by ADP) also induces microglial pro-inflammatory cytokine release, upregulating excitatory synaptic transmission in the dorsal horn and participating in neuropathic pain [112,114,115,116]. Moreover, these P2 receptors can be activated by the release of ATP by spinal astrocytes, promoting cytokine production and a pro-inflammatory milieu [117]. 

*P2X7 receptor*. P2X7 receptor functions as an ion channel, but sustained stimulation with large amounts of extracellular ATP induces its conformation as nonselective large pores in cell membrane. It is present in microglia and plays a role in the maintenance of neuropathic pain. Specific inhibition of P2X7 receptor pore formation, without affecting its cation channel activity, reduces chronic pain [118].

In microglia, the activation of P2X7 receptor causes the release of the enzyme Cathepsin S (CatS). Extracellular CatS splits the chemokine domain of neuronal membrane-bound fractalkine (FKN) present in dorsal horn neurons. Soluble-FKN (s-FKN) interacts with microglia CX3C chemokine receptor 1 (CX3CR1), stimulates p38/MAPK, and promotes the release of cytokines that sensitize the second order neurons of the sensory pathway [119,120,121]. Liu et al. showed an increase in the expression of FKN/CX3CR1/p38/MAPK and in the amount of microglia in the dorsal horn of the sciatic nerve, in an endometriosis rat model [122]. This microglia–nerve crosstalk plays a role in central sensitization and could explain one of the mechanisms associated with hypersensitivity and allodynia in endometriosis. Moreover, increased levels of s-FKN were found in the peritoneal fluid of women with endometriosis [123]. In the ectopic endometrial lesions of a sciatic endometriosis model in rats, increased levels of FKN and s-FKN as well as CX3CR1 have been detected, with a positive correlation with the severity of hyperalgesia [122]. In ectopic lesions, membrane-bound FKN was found in the macrophage surface, whereas CX3CR1 was present in nerve fibers, specifically in Schwann cells. As previously noted, macrophages express P2X7 receptor, which could be an essential piece in FKN cleavage in the endometriotic foci. s-FKN can act as a CX3CR1 ligand as well as being a potent chemoattractant that would favor the development and maintenance of the inflammatory microenvironment in the ectopic lesion, mainly around nerve fibers. On the other hand, a direct interaction between macrophage membrane-bound FKN and CX3CR1 of Schwann cells has been suggested, which could cause myelin phagocytosis and activation of Schwann cells involved in peripheral sensitization in ectopic lesions. Therefore, P2X7 receptor could contribute to peripheral and central hypersensitivity in endometriosis [122].

Although microglial P2X7 receptor activation by presynaptic neurons is mainly related to the release of pro-inflammatory cytokines, it also causes more ATP release into the dorsal horn microenvironment. This is made possible by the increase in the intracellular calcium resulting from P2X7 action that activates pannexin-1. The increasing extracellular ATP levels potentiate purinergic signaling in pain effectors and induce chronification of pain [124]. 

#### 4.1.6. Adenosine and Adenosine Receptors (AR): Analgesic and Anti-Inflammatory Effects

Adenosine, the hydrolysis product of purine nucleotides such as ATP and ADP, often plays the opposite role to them, leading to a compensatory system in physiological and pathological conditions. In fact, endogenous adenosine has modulating effects on neuronal and glial cells with implications in pain transmission. For this reason, adenosine has been proposed as a potential analgesic target for nociceptive, inflammatory, and neuropathic pain. Therefore, the adenosine signaling pathway may be an interesting target to treat endometriosis-associated pain. However, determining the effects of adenosine in endometriosis-associated pain is quite complex. It has been experimentally proven that adenosine can produce antinociceptive and anti-inflammatory effects as well as their opposite, depending on the site of action, the receptor activated, the extent of exposure, and the context. 

Although the role of P1 receptors in endometriosis-associated pain has not yet been studied, we briefly outline the different effects that have been described in the literature and their possible usefulness for the treatment of endometriosis-associated pain.

*A_1_ adenosine receptor (AR)*. A_1_AR is the main adenosine receptor associated with inhibitory neuromodulation of pain. A_1_AR regulates neurotransmitter release, neuronal excitability, and pain reduction. This high adenosine affinity receptor is expressed at peripheral sensory nerve endings [125], in dorsal horn neurons of the superficial layers of spinal cord [126], and in microglia [127].

A_1_AR is G_i_-coupled and its activation inhibits AC activity (inhibition of cAMP production), which leads to hyperpolarization by increasing potassium conductance, blocks transient calcium channel opening, and stimulates PLC, inducing an increase in inositol 1,4,5-triphosphate (IP_3_) and intracellular Ca^2+^ levels and stimulation of calcium-binding proteins such as protein kinase C (PKC) [128]. Furthermore, A_1_AR presynaptically inhibits primary sensory neuron transmission onto dorsal spinal neurons by blocking neurotransmitter release [126,129]. Moreover, when primary sensory afferent neurons depolarize, afferent nerve terminals release glutamate and SP, but also adenosine, in the dorsal spinal cord [129]. In this situation, adenosine can act as a negative modulator by activating A_1_AR at postsynaptic sites.

As mentioned, A_1_AR is also present in spinal cord microglia where it is upregulated by high levels of extracellular ATP. The activation of A_1_ARs curbs activation of microglia and blocks their role in neuronal sensitization [127]. 

Several clinical studies have demonstrated that specific A_1_AR stimulation results in analgesic effects sufficient to ameliorate nociceptive, neuropathic, and inflammatory pain (reviewed in [18,130]). This antinociception, and specifically the reduction of hyperalgesia in several pain models, suggests A_1_AR agonists as possible tools for treating endometriosis-associated chronic pain. 

*A_2A_AR.* A_2A_AR is present in immune cells, neurons, and glial cells, among other cell types. In fact, A_2A_AR is considered a potential therapeutic target in treating chronic pain of neuroinflammatory origin. A_2A_AR action is, however, controversial due to an apparent dichotomy between peripheral and central signaling.

At the molecular level, A_2A_ARs are directly coupled to G_s_ intracellular proteins, leading to the activation of AC (activation of cAMP production) with the consequent activation of the protein kinase A (PKA) and PKC signaling cascade. Increased cAMP levels in primary afferent neurons contribute to peripheral sensory nerve stimulation and increased excitatory neurotransmitter release in the spinal cord, enhancing nociception [131]. 

In addition, A_2A_AR is considered the main mediator of anti-inflammatory responses through its expression in a wide range of immune cells. A_2A_AR activation increases intracellular immunosuppressive cAMP in peripheral immune cells. This may contribute to reducing inflammatory pain by modulating these cells and consequently decreasing the levels of sensitizing substances (e.g., inflammatory cytokines from macrophages or histamine from mast cells) in the endometriotic microenvironment [130]. 

In addition, A_2A_AR modulates immunoresponses of microglia. As in peripheral immune cells, A_2A_AR agonists produce a cAMP signaling cascade that attenuates pro-inflammatory cytokine production and increases anti-inflammatory cytokine release, mainly IL-10. The reduction of the inflammatory state relieves neurophatic pain [132,133,134,135]. 

Conversely, under pathologic conditions, A_2A_AR activation is also related to a pro-inflammatory role [136,137]. During neuropathic pain, persistent activation of spinal microglia occurs. The sustained release of stimulating molecules such as BDNF in spinal cord exacerbates neuronal hypersensitivity and chronic neuroinflammation. As noted above, ATP mediates BDNF release via P2X4 receptor from activated microglia, as well as by adenosine via A_2A_AR. Constitutive release of BDNF is under the control of PKC, but, in some pathological conditions, upregulation of A_2A_AR in microglia has been detected. The high levels of intracellular cAMP and the subsequent activation of PKA prevails over PKC actions inducing increased BDNF release [137]. However, the increase in A_2A_AR levels in microglia is mainly described in brain with chronic neuroinflammation in cases with neurodegenerative diseases, where A_2A_AR interaction with several neurotransmitters plays an important role in pathology progression [138]. Further studies are needed to determine if A_2A_AR upregulation in activated microglia also occurs in endometriosis-associated pain. If that is the case, the therapeutic strategies involving A_2A_AR activation might not be good for the evolution of chronic pain related with endometriosis. 

In conclusion, therapeutic tools that potentiate A_1_AR-mediated antinociceptive and A_2A_AR-mediated anti-inflammatory actions of adenosine could have analgesic effects that would be useful for the management of endometriosis-associated pain. 

*A_2B_AR*. A_2B_AR is mainly present in immune cells, as well as, at low levels, in spinal cord and CNS, and prominently in astrocytes [18,19]. Its ubiquitous expression in inflammatory cells suggests a role in inflammatory pain through the release of various inflammatory mediators.

A_2B_AR has a low affinity for adenosine, and it is therefore significantly activated under pathophysiological conditions, when adenosine concentration is high. Similar to A_2A_AR, A_2B_AR activates AC, leading to a PKA phosphorylation cascade. This cellular signaling mainly promotes an anti-inflammatory response in immune cells and plays a part in the attenuation of acute inflammation [128]. 

While it may appear that A_2B_AR plays the same role as A_2A_AR, its effect is sometimes opposed. This is possible because, as with A_1_ and A_3_AR, A_2B_AR is coupled to PLC, leading to the accumulation of intracellular Ca^2+^, the generation of IP_3_ and diacylglycerol (DAG), and the activation of PKC, involved in the release of pro-inflammatory mediators such as IL-6 from macrophages and IL-1β and VEGF from mast cells, with indirect effects on inflammatory pain [19,130,139]. In fact, Hu et al. described a mechanism underlying A_2B_AR-mediated chronic pain in three independent models of chronic pain [140]. The prolonged elevated adenosine levels activate A_2B_ARs of myeloid cells. This activation increases the production and release of IL-6 and soluble IL-6 receptor (sIL-6R). The complex IL-6/sIL-6R trans-activates gp130 on primary sensory fibers, which in turn stimulates the transcription factor pSTAT3, inducing TRPV1 gene transcription. The increase of TRPV1 in nociceptors produces sensory neuron hyperexcitability and can produce chronic inflammatory pain. 

These opposing effects of A_2B_AR hamper the use of its agonists or antagonists as potential therapeutic agents for inflammatory disorders and pain. Evaluating the possible use of A_2B_AR as therapeutic target of endometriosis-associated pain requires a better understanding of the function of A_2B_AR in this disease. Moreover, the loss of expression of some ectonucleotidases in endometriotic tissue and the large amount of ADA in the fluid contents of endometriomas suggests that the adenosine levels in endometriotic milieu are low, rendering activation of A_2B_ARs unlikely. Consequently, A_2B_AR does not seem to be a suitable target for endometriosis treatment.

*A_3_AR*. A_3_ARs are described in peripheral and central neurons, glial cells, and immune cells [141,142]. Over the last decades, experimental studies with A_3_AR agonists showed the attenuation of nociception and neuropathic pain [130,141,142,143]. This has made A_3_AR a focus of development of new therapeutic strategies for pain.

Similar to A_1_AR, A_3_AR is coupled to G_i_, which inhibits AC, and to G_q_, which activates PKC. On primary sensory neurons, A_3_AR inhibits the Ca^2+^-dependent K^+^ currents, reducing their excitability and neurotransmitter release [142]. This mechanism exerts an antinociceptive effect, but it is not the only one. 

Agonists of A_3_AR enhance and recover inhibitory GABA signaling in spinal cord neurons. In neuropathic pain states, reduced levels of spinal GABA and GAD65 [144,145], its synthesis enzyme, are detected, whereas the expression of GABA transporter GAT-1, which uptakes the neurotransmitter from the synapse, is increased [145]. Additionally, there is a reduction in the activity and expression of KCC2. As noted above, P2X4 receptor activation promotes the production and release of BDNF, which, via TrkB, downregulates KCC2 and disrupts Cl^-^ homeostasis, altering GABA_A_ receptor postsynaptic inhibitory control and leading to neuronal hyperexcitability [109,110,111]. A_3_AR activation reverses these situations. The use of A_3_AR agonists maintains the phosphorylation status of GAD65 and GAT-1, which stabilizes and activates GAD65 and leads to the internalization and inactivation of GAT-1 [146]. In addition, A_3_AR agonists enhance KCC2 phosphorylation, increasing its activity [146]. Therefore, the activation of A_3_AR allows the increase of GABA levels and the restoration of inhibitory actions of GABA in the spinal cord, alleviating neuropathic pain. Moreover, A_3_AR activation also diminishes the production of pro-inflammatory cytokines (TNF-α and IL-1β) and enhances the formation of anti-inflammatory cytokines (IL-10) in the spinal cord by inhibiting the p38/MAPK and NF-_kB_ signaling pathways [143,147,148]. This enhances the reduction of glial activation, decreasing neuroinflammation and pain hypersensitivity.

In recent years, antinociceptive properties of A_1_AR and A_2A_AR agonists have been the most studied targets of adenosine signaling to relieve acute and chronic pain. However, A_3_AR agonists have increased in importance in pain treatment since they avoid the undesirable cardiovascular side effects of A_1_AR and A_2A_AR agonists [149,150]. For this reason, the use of selective A_3_AR agonists seems promising as a safe and effective analgesic treatment for chronic pain, and it is also being considered to treat endometriosis-associated pain. 

## 5. Perspectives of ATP and Adenosine Signaling Modulation: Possible Tools to Treat Endometriosis-Associated Pain

Most medical treatments for endometriosis are aimed at relieving the chronic pain associated with the disease, but in many cases endometriosis-associated pain symptoms are not improved, and, if they are, they return with treatment cessation (reviewed in [53]). 

As we try to document in this review, there is clear involvement of purinergic signaling in the generation and modulation of the sensation of pain. ATP participates in the hyperexcitability of sensory neurons and the development and maintenance of different types of pain (nociceptive, inflammatory, and neuropathic). Evidence mostly suggests ATP is responsible for triggering nociceptive pain, as well as inflammatory responses in the body, in contrast to adenosine. ATP also influences fecundation, endometrial receptivity, and embryo implantation [2].

In endometriosis, the adhesion of viable endometrial cells to establish an ectopic endometriotic focus triggers signals of injury and promotes the activation of an immune system that is inefficient in clearing ectopic cells. This situation is reflected in a significant increase in extracellular ATP levels in the endometriotic lesion. The ATP-rich microenvironment of the endometriotic lesion contributes to the two main symptoms of endometriosis: pain and infertility. The increase in ATP levels is enhanced by the loss of expression of certain ectonucleotidases in endometrial ectopic cells, prolonging signaling and activating pain. It seems, then, reasonable that development of therapeutic approaches for endometriosis-associated pain be based on achieving a decrease in ATP levels and their signaling cascade, and/or increased adenosine levels and activation of their receptors. Despite the potential of purinergic-based drugs and their analgesic effects in various pain models, there is no medication presently available for clinical use.

Currently, most pharmacological therapies for endometriosis are hormonal therapies. Neither these nor surgical treatment are compatible with the pregnancy desire of women with endometriosis, and it is usually a matter of choosing between alleviating symptoms and getting pregnant. A non-hormonal alternative to alleviate endometriosis-associated pain may lie in purinergic-based drugs. The development of clinical drugs targeting purinergic receptors is not free of difficulties, such as the ubiquitous expression and wide action of purinergic receptors throughout the body, and the large number of receptor subtypes combined with a lack of complete knowledge of their physiological and pathophysiological functions.

We present below a selection of potential purinergic signaling targets for pharmacological treatment of endometriosis-associated pain, without ruling out their utility in the improvement of the fertility of these women.

**(I)**  **P2 Receptor Antagonists**

ATP, through P2X and P2Y receptors, induces cell signaling directly involved in hyperexcitability of sensory neurons and sustained glial cell reactivity in neuropathic pain. P2 receptor antagonists and other molecules that alter their function have been used to describe the involvement of ATP in pain signaling. Moreover, the attenuation of nociception, hyperalgesia, and allodynia by blocking some P2 receptors suggests that P2 receptor-related drugs are potential candidates for the treatment of chronic pain conditions. In fact, P2X3 receptors of sensory neurons seem to be the main receptors involved in pain, and P2X4 and P2X7 receptors appear to be key elements in neuropathic and inflammatory pain for their function in glial and immune cells.

P2X3 receptor is involved in the development and progression of endometriosis-associated pain [81,82,86,87], and there is a growing interest in the development of substances to interfere or inhibit its function. A P2X3 antagonist, MK-7264/AF-219, known as gefapixant, is currently being tested in various advanced clinical trials with subjects with pulmonary disease and chronic cough (NCT01432730, NCT02502097, and NCT02477709). Recently, a clinical trial has been begun to evaluate the efficacy and safety of gefapixant in women with endometriosis-associated pain (NCT03654326). Experimental evidence of the relief of endometriosis-related pain has been obtained with the use of P2X3 receptor antagonists (e.g., A-317491), in endometriosis-induced animal models [87]. 

The use of P2X4 receptor antagonists (such as CORM-2, 5-BDBD, and NP-1815-PX) and P2X7 receptor antagonists (such as AZD9056 and AZ11645373) has been reported to inhibit microglia activation, significantly reducing inflammation and alleviating pain (reviewed in [151,152]). Although the administration of AZD9056 yielded good results in pain models in animals, clinical trials were not successful in alleviating symptoms in patients with rheumatoid arthritis, a chronic inflammatory disorder [153]. This suggests that new pharmacological strategies with P2X4 and P2X7 as targets are needed to achieve the desired clinical results in the treatment of inflammation and pain in endometriosis.

P2Y receptors are also potential pharmacological targets. For example, P2Y_12_ receptor regulates microglial activation and the neurotransmission of the excitatory signal in spinal cord neurons; the administration of P2Y_12_ antagonists blocked microglia action in nerve injury-induced pain models [115,154]. There are clinical trials with P2Y_12_ antagonists, in cardiovascular pathologies, but not yet in pain. However, the antithrombotic actions of P2Y_12_ antagonist drugs could certainly complicate their use.

**(II)**  **The Control of ATP Release**

ATP release can occur via vesicular and non-vesicular mechanisms. Targeting ATP release on the endometriotic lesions, on sensory and central neurons, would represent a fine-tuning regulation of the P2 receptors involved in pain. 

Vesicular ATP release involves the mechanism of exocytosis. Vesicular nucleotide transporter (VNUT), encoded by *SLC17A9* gene, is responsible for the vesicular storage and release of ATP in neurons, astrocytes, and microglia [155,156]. It is known that secretion of ATP through VNUT-dependent vesicular release mechanisms is involved in purinergic signaling in pain and inflammation [155]. 

After peripheral nerve injury, increases in VNUT expression and extracellular ATP levels are detected in spinal cord [156]. Masuda et al. showed that mice lacking VNUT in the dorsal horn neurons reduced the alloydina evoked by peripheral nerve injury. This did not occur with mice lacking VNUT in primary sensory neurons, astrocytes, or microglia. Increased extracellular ATP levels and neuropathic pain were restored in these mice lacking VNUT in dorsal horn neurons when VNUT expression was restored [156]. 

Clodronate and etidronate are biphosphonates used in osteoporosis therapy that have analgesic properties. In vitro assays demonstrated that they inhibited VNUT, leading to the modulation of ATP release and purinergic transmission [157,158]. In addition, in vivo studies with clodronate showed attenuation of neuropathic and inflammatory pain [158]. As clodronate is approved for clinical use in the treatment of osteoporosis and its safety is proven, it may be a good candidate for the treatment of endometriosis-associated pain.

ATP release in neurons, astrocytes, and microglia also occurs through membrane channels, such as pannexin hemichannels, connexins, and the P2X7 receptor itself. In recent years, a wealth of evidence has pointed to pannexin-1 [124,159,160] and connexin-43 (reviewed in [161,162,163]) as crucial elements in the induction and maintenance of chronic pain. These ATP-permeable channels are, therefore, pain relief targets for further investigation.

**(III)**  **Recombinant Ectonucleotidases**

In endometriosis, a loss of ectonucleotidase expression is reported in association with the severity of the disease. This might contribute to a rise in ATP levels. Restoring the ectonucleotidase activity in the endometriotic tissue would consequently reduce ATP-induced pain and enhance the antinociceptive effects of adenosine in the pain pathway. 

CD39, CD73, and prostatic acid phosphatase (PAP) have been described as the main ectonucleotidases involved in the production of adenosine in DRG and spinal cord neurons [128,164]. A single intrathecal injection of recombinant soluble CD73 or PAP had long-lasting antinociceptive effects, dependent on A_1_AR activation, including antihyperalgesic and antiallodynic effects, in naïve mice and in mouse models of inflammatory and neuropathic pain [165,166].

Recombinant ectonucleotidases are potential tools for endometriosis-associated pain treatment. Additional preclinical and clinical studies are required to confirm their benefit in inflammatory and neuropathic pain.

**(IV)**  **P1 Receptor Agonists**

Adenosine has a limited use due to its short life in vivo. Alternatively, agonists and positive allosteric modulators of AR have been described as pharmacological tools to treat inflammation and pain (reviewed in [167,168,169]). Their analgesic and anti-inflammatory effects have been studied with A_1_AR, A_2A_AR, and A_3_AR agonists. The controversial role of A_2B_AR limits their therapeutic use. 

Several preclinical and clinical trials with agonists of A_1_AR (e.g., GW493838 and NCT00376454) and A_2A_AR (e.g., BVT.115929 and NCT00452777) have been performed. Despite the good results of AR agonists in several pain models [126,132,133,134,135], the lack of analgesia in humans together with the evidence of undesirable side effects, such as cardiovascular involvement, jeopardizes their use in any therapy [168,169]. 

On the other hand, preclinical and human clinical studies with A_3_AR agonists did not have significant side effects [170]. Antinociceptive effects have been described with the use of moderately selective agonists of A_3_ARs, such as IB-MECA and Cl-IB-MECA, and highly selective agonists, such as MRS5698 and MRS5980. In general, A_3_AR agonists tend to restore the altered pain signaling of chronic pain. For example, A_3_AR agonists decrease glial activation and the generation of pro-inflammatory cytokines [143,147,148], increase the production of anti-inflammatory cytokines [147,148], and restore the inhibitory action of GABA [146]. Interestingly, A_3_AR agonists selectively modify pathological pain but seem not to alter protective pain [171]. Phase II and III clinical trials with IB-MECA have been completed in rheumatoid arthritis (NCT01034306) and psoriasis (NCT01265667), respectively. A phase II clinical trial with Cl-IB-MECA was also completed in chronic hepatitis C (NCT00790673). Moreover, Cl-IB-MECA is currently in phase II for its antitumor effects in hepatocellular carcinoma (NCT02128958). Although these clinical trials do not attend to their effectiveness in chronic pain, the safety profile in chronic inflammatory diseases, liver disease, and cancer envisages an optimistic future in the pharmacological treatment of neurophatic endometriosis-associated pain. 

Several studies have demonstrated that acupuncture improves endometriosis-associated pain [54,172,173]. Interestingly, mechanisms underlying acupuncture-induced analgesia involve purinergic signaling [174,175,176]. Acupuncture produces local release of ATP leading to the activation of purinoreceptors on sensory nerve endings, triggering the neurotransmission of pain signal to brain. Local release of adenosine in certain centers of the brain cortex can modulate and inhibit pain sensation through the activation of AR, mainly A_1_AR [175,177]. Thus, acupuncture is a possible complementary treatment to relieve endometriosis-related pain.

In addition, the potential analgesic effect of adenosine receptor antagonists such as caffeine has also been studied. Caffeine, at low doses, and in combination with analgesic drugs, acts as an adjuvant (reviewed in [178]). At dietary levels, caffeine has a high affinity for A_1_, A_2A,_ and A_2B_ARs [179]. Adenosine-based mechanisms involved in caffeine pharmacological antinociception are attributed to the A_2A_ and A_2B_AR blockade [178]. Despite its auspicious effects, caffeine has not been tested in endometriosis-associated pain. In fact, we predict that the use of caffeine in relieving endometriosis-associated pain is a complex matter due to the role of caffeine in female hormone pathways, in turn influencing the endometriosis outcome [180,181,182]. Moreover, no consistent association has been found between coffee/caffeine intake and the risk of this hormone-dependent disease [183,184,185]. Furthermore, the inhibition of A_1_AR by caffeine might interfere with the effectiveness of several analgesic agents and treatments, e.g., acupuncture (reviewed in [178]), which should be taken into account in the clinical management of patients.

**(V)**  **Inhibitors of Equilibrative Nucleoside Transporters**

In contrast to ATP, adenosine is neither stored nor released in synaptic vesicles. However, adenosine can be released by the cell via nucleoside transporters. One way to increase local extracellular adenosine levels and the concomitant antinociceptive signaling is the blocking of the equilibrative nucleoside transporters (ENTs). ENTs regulate facilitated diffusion and bidirectional nucleoside transport across the cell membrane, following the concentration gradient, in a number of tissues, including central nervous system. ENT-1 is highly expressed in superficial dorsal horn laminae and in DRG, colocalizing with A_1_ and A_2_AR [186,187]. 

Maes et al. demonstrated that systemic administration of ENT-1 inhibitors can reverse hyperalgesia in guinea pig inflammatory pain models [188]. The mechanism underlying this analgesia seems to be the blocking of adenosine reuptake into cells, allowing greater activation of A_1_ and A_2_AR [188]. These results show that it is necessary to investigate the potential therapeutic effect of ENT inhibition. Moreover, the use of ENT inhibitors in combination with AR agonists or ADA inhibitors might enhance the antinociceptive effects of these molecules. 

Consequently, the use of ENT inhibitors for analgesia in endometriosis is worth studying.

**(VI)**  **Inhibitors of AK and ADA**

Adenosine metabolism is mainly the responsibility of AK and ADA enzymes; their inhibition would result in an increase in local extracellular adenosine levels, enhancing their antinociceptive signaling. 

Several studies have shown that the supply of an orally active non-nucleoside AK inhibitor (ABT-702) produced effective antinociceptive and anti-inflammatory effects both in vitro and in vivo [189,190,191]. Increased adenosine concentration by AK inhibition seems to produce therapeutic effects through the activation of A_1_ and A_2A_AR [192]. Moreover, its pharmacological action is achieved with lower doses than with AR agonists, thus reducing the probability of producing psychomotor and cardiovascular side effects. Nevertheless, other AK inhibitors have toxic side effects, such as neurotoxicity (reviewed in [192]). The findings about ABT-702 indicate this to be an efficient and safe drug for the treatment of neuropathic pain and inflammatory states. Studies of the effects of ABT-702 in induced-endometriosis animal models are needed to assess its utility in therapies against endometriosis-associated pain.

It is notable that ADA, another metabolic enzyme of adenosine, and AK have different kinetics: with low or moderate inflammation, AK is the one modulating adenosine levels; with greater inflammation, where there is a substantial elevation of adenosine levels, ADA activity gains importance [193]. Hence, the effect of their inhibitors would also vary depending on adenosine levels in the environment. While the antinociceptive effect of AK inhibitors has been demonstrated, inhibition of ADA does not produce an intrinsic antinociceptive effect without very high levels of adenosine [194]. This is why the use of ADA inhibitors is only considered as an enhancer of the antinociceptive effects of AK inhibitors [195]. ADA inhibitors, such as deoxycoformycin, have been shown to have anticancer effects and to be useful in the treatment of infectious diseases [196,197,198]. Unfortunately, these compounds are usually toxic at effective doses [196,198,199,200]. The co-administration of AK inhibitors, such as ABT-702, with ADA inhibitors at lower doses as adjuvants, seems to be a suitable strategy for analgesia in endometriosis patients, but exhaustive prior safety studies are required. 

## 6. Conclusions

Pain has a strong impact on the quality of life of women with endometriosis. Current surgical and pharmacological treatments for endometriosis have as their primary goal the relief of pain. Nevertheless, these treatments have a limited success rate and in general hamper pregnancy. Although there is increasing understanding of the essential role of purinergic signaling in the development and progression of nociceptive, inflammatory, and neuropathic pain, its implication in endometriosis-associated pain is still poorly studied. In this review, we examine the role of purinergic signaling, and particularly the role of extracellular ATP as a triggering factor for acute and chronic pain signaling, in the context of endometriosis.

Ectonucleotidases, the enzymes regulating ATP levels in the extracellular milieu, are altered in endometrial tissue in endometriosis. Of note is the decrease in the expression of the CD39–CD73 axis that supports the hypothesis of ATP (rather than adenosine) accumulation. Concomitant sustained activation of P2 receptors, capable of generating a persistent pain message, is compatible with the onset and maintenance of endometriosis-associated pain. It is known that P2X3 receptor, expressed in sensory neurons, mediates nociceptive, neuropathic, and inflammatory pain, and it is enrolled in endometriosis-related pain; therefore, pharmacological P2X3 inhibition is a worthy candidate for testing; in this sense, the use of the P2X3 receptor antagonist gefapixant is under clinical study. Although the P2X3 receptor fulfills the requirements to be a suitable molecule to be targeted, other ATP receptors have to be considered as well, such as the P2X4 and P2X7 receptors that are involved in macrophage–nerve and microglia–nerve interactions, promoting a persistent inflammatory state and the chronification of pain. P2X4 receptor triggers the generation of the inflammatory pain sensitization mediator PGE2 and is involved in the decrease of inhibitory control of GABAergic interneurons in neuropathic pain. In contrast, P2X7 receptor promotes the maintenance of neuropathic pain through the FKN/CX3C/CX3CR1 pathway, which seems to be altered in endometriosis. The use of P2X4 and P2X7 antagonists has yielded good results in reducing inflammation and alleviating pain in animal models. However, a clinical trial with the P2X7 receptor antagonist AZD9056 has been unsuccessful in a chronic inflammatory disorder, rheumatoid arthritis. 

P2Y_1_ and P2Y_2_ receptors are involved in the activation and regulation of P2X3 and TRPV1 receptor ion channels of nociceptors. Moreover, microglial P2Y_12_ and P2Y_13_ receptors activation triggers the release of pro-inflammatory cytokines, increasing excitatory synaptic transmission in the dorsal horn. Although P2Y receptors are involved in the modulation of pain, there are no clinical trials evaluating the use of P2Y antagonists in pain.

In addition, the antinociceptive and anti-inflammatory actions of the ARs, mainly A_1_AR and A_3_AR, have encouraged studies using agonists of these receptors for the treatment of pain and chronic inflammatory diseases. With the current knowledge, A_3_AR-targeting drugs are potential tools to treat neuropathic pain, as well as endometriosis-associated pain, but clinical studies in this regard are needed. 

In summary, purinergic signaling-based strategies need to be further explored in the medical management of endometriosis-associated pain. This will also improve treatment of other symptoms of endometriosis, such as infertility, in which purinergic signaling also plays a role.
